# Cases of Ligature of the Common Carotid Artery

**Published:** 1834-01-01

**Authors:** Herbert Mayo

**Affiliations:** Surgeon to the Middlesex Hospital, &c.


### Cases of Ligature *of the* Common Carotid Artery. By Herbert Mayo, F.R.S., Surgeon to the Middlesex Hospital, &c.

I.

In the number of the London Medical and Physical Journal for November, 1828, a case is described, in which I tied the common carotid artery for repeated arterial hemorrhage from a sloughing ulcer of the pharynx. After the carotid had been tied, the hemorrhage did not return, and the patient was shortly restored to health. But in two years the same person fell into a decline: he was admitted again into the Middlesex Hospital, in the last stage of phthisis, in the summer of 1833. Upon his death, the neck was examined; and the artery which had bled was ascertained to be the lingual. This was in accordance with the conjecture, which I had formed as to the source of the hemorrhage at the time of tying the common carotid. In a case, which I had previously seen with Dr. Watson, of bleeding into the fauces after cynanche tonsillaris, and which terminated with the patient being suddenly suffocated by blood pouring down the windpipe, the ulcerated artery was likewise the lingual. These facts, coupled with the exposed situation of the lingual artery towards the fauces, render it probable, that in the majority of cases of bleeding from the side of the pharynx, the lingual artery is the source of the hemorrhage.

It may likewise be inferred from the preceding case, taken with another, which will be narrated in the present paper, that ligature of the common carotid is sufficient permanently to restrain hemorrhage from the branches of the external carotid, when one or more of the latter happen to have been opened by ulceration, or in wounds. But this operation can only be safely recommended in young persons, and in persons free from deranged circulation in the head. In persons past the age of fifty, or labouring under cerebral excitement, it is probably unsafe to disturb the circulation in the brain to the extent which must follow the obliteration of one of the carotid arteries; and I should be rather disposed, on the recurrence of such a case, to cut down upon the external carotid, and to tie it immediately below the origin of the lingual; tying at the same time the upper thyreoid half an inch from its origin. The objection to this operation is, that it is more tedious and difficult than that of tying the common carotid. And peculiar circumstances may of course be present, as in Case III., which may render the ligature of the common carotid advisable on other grounds.

II.

James Blackett, a remarkably fine infant, of the age of five months, was admitted an out-patient of the Middlesex Hospital, in August last. There was a considerable and increasing swelling on the left side of the face, which extended from the level of the upper edge of the ear to below the jaw, and from the tragus, which it pushed backwards, half way to the chin. At two or three points the skin covering the swelling was slightly raised, with a shining and irregular and highly vascular surface; in other words, there were several vascular nævi, or aneurysms by anastomosis, upon the skin covering the swelling; and the swelling itself consisted of a subcutaneous vascular tumor, continuous with the superficial nævi. The tumour grew into the ear: the fore part of the external passage of the ear was red and prominent, encroaching into and narrowing the meatus. When the child coughed or cried, the tumour became more full and swollen.

The extent and probable depth of the tumour rendered it impracticable to include it in ligatures, and made it likely that, if excision was attempted, the little patient would die of hemorrhage.

Under these circumstances, the practice which I adopted consisted in passing four double threads, or small setons, in different directions through the tumour. No bleeding followed the introduction of the threads. In a few days there was a free discharge from each seton. The threads were not removed till the expiration of a month. The tumour had undergone no diminution, but had become fuller at the upper part, where the skin had already begun to ulcerate. The surface ulcerated was of the size of a sixpence.

A week after the removal of the setons I tied the common carotid artery. The operation was followed by no ill consequence. The ligature came away on the eighth day. The effect of the operation on the tumour was decided and satisfactory. The ulcer at the upper part healed, the tumour flattened, and in a fortnight was reduced a third in volume. A week afterwards pressure was made upon the cheek, by means of a piece of lead attached by adhesive plaster. The pressure has been continued to the present time: it has excited a return of discharge from the setons on the cheek which had closed. The tumour on the cheek has continued to diminish, and is externally less by one half than at the time of the operation.

It is now two months since the carotid was tied. What the final result of the treatment employed in this case will be is yet uncertain.

The operation of tying the carotid in this infant was extremely difficult, owing to the shortness, fulness, and vascularity of the neck, and the delicacy of the parts, which made me fearful of injuring the internal jugular vein, in passing the ligature round the vessel.

In placing a ligature round the carotid artery, it is always more convenient and safer to pass the aneurysmal needle from the inside of the vessel, and to bring it out between the artery and vein, than to introduce it between the two.

III.

A gentleman, ætat. thirty, suffered under the severest mental anguish, which bordered upon distraction, and excited the fears of his friends for his safety. Placed under proper medical care, he became seemingly more tranquil; but, when the vigilance of his attendants relaxed, he made a desperate effort to destroy himself: he stabbed himself in the right side of the throat, immediately below the angle of the jaw, with a penknife, which penetrated a depth of four inches, and divided the external carotid above the origin of the lingual artery. This, however, was learnt only after the fatal termination of the case. At the time there was a great effusion of blood, and he became insensible. When he was roused from this state, the hemorrhage did not recur.

I first saw this patient eight days after the attempt at self-destruction, up to which period every thing had gone on favourably. A sudden gush of arterial blood had now taken place from the wound. When about a pint of blood had been lost, the patient became faint, and the hemorrhage stopped. The wound of the integument was three quarters of an inch in length, and irregular, showing that he had made repeated pushes with the knife, after he had forced it into his neck. I removed the clot from the wound. The patient was raised; he swallowed some liquid; coughed; the bleeding did not recur. The finger, introduced into the wound, passed a considerable depth, and seemed to extend behind the upper part of the pharynx. It was of course impossible to conjecture with certainty what artery had been wounded: it was even conceivable, from the direction of the wound, that the internal carotid had been injured. The patient's neck was naturally short and full, and the parts around the wound were swollen, and condensed by inflammatory infiltration.

As the smallness of the external wound rendered it easy to arrest hemorrhage from it, I determined to wait, and to avail myself of the chance that the hemorrhage might not recur. I therefore dressed the wound lightly, and left a person with the patient capable of arresting the bleeding, if it returned. Six hours afterwards the hemorrhage broke out anew; it was suppressed in a minute, but nearly a pint of blood had been lost. There was now too great a certainty that the bleeding would recur, if nothing were done to prevent it: I therefore tied the common carotid artery in the middle of the neck.

The patient went on favourably for a few days; but he exhibited at times considerable excitement, and he complained of pain at the upper and back part of the head. When the head was tapped at this part, he felt the pain shoot through the head. Leeches were applied to the temples, and cold, and the bowels were repeatedly acted on by medicine.

In the evening between the sixth and seventh day after the operation, two or three ounces of blood were observed to have escaped from the wound made in tying the artery. Six hours afterwards a violent gush of blood took place from the carotid, which had ulcerated below the ligature. The instantaneous faintness of the patient, and pressure made by the attendant, stopped the hemorrhage. Being sent for, I enlarged the wound, and applied another ligature below the first, which had not separated from the artery. The patient complained of numbness of the left hand. He said that, when he was bleeding, he felt his left arm become numb, and supposed that it was death beginning. The numbness of the arm and loss of power in it, increased day by day; then the left leg became numb and powerless, and the left side of the face became partially affected. He became violently delirious, and sank, and died on the sixth day after the second operation.

Upon examining the head and neck after death, matter and lymph were found in a thick layer between the arachnoid and pia mater, covering the upper and back part of the right hemisphere of the brain; and at two parts the matter formed considerable abscesses between the convolutions, which were pressed aside, and their texture softened, and partially absorbed to give room for the matter. One abscess was of the size of a bullet, the other as large as a chesnut.

The ulceration of the artery behind the ligature was in part the result of a general tendency to suppuration in the system, evinced by the formation of abscess in the brain. But, besides this cause, it is to be surmised that little or no clot had formed in the artery below the ligature. A strongly adherent clot, nearly an inch in length, had formed below the second ligature. The secondary hemorrhage from the carotid occurred on the sixth day after the vessel had been tied. In a patient in whom I tied the external iliac artery for femoral aneurism, in September last, secondary hemorrhage, from ulceration behind the ligature, supervened before the completion of the seventh day. In this case, to suppress the bleeding, I was compelled to tie the common iliac. The patient did not survive beyond twenty hours; but a large and sufficient clot had already formed in the common iliac, behind the ligature.

It is evident that, after tying an artery, all the conditions should be most carefully promoted, which encourage the coagulation of the blood. On examining the arteries in patients who have died after amputation, they are generally found to contain a clot: but this is not always the case. Secondary hemorrhage from arteries which have been tied on a stump is proportionally rare, although it occasionally occurs. In one case, in which violent arterial hemorrhage supervened ten weeks after amputation above the knee, through a sinus which led to a portion of exfoliating bone, I found it necessary to tie the femoral artery at the groin. The patient did well.

When an artery has been tied, there is danger of secondary hemorrhage from the fifth day to the period, however distant, of the entire closing of the wound.

